# The True Value of HbA1c as a Predictor of Diabetic Complications: Simulations of HbA1c Variables

**DOI:** 10.1371/journal.pone.0004412

**Published:** 2009-02-11

**Authors:** Marcus Lind, Anders Odén, Martin Fahlén, Björn Eliasson

**Affiliations:** 1 Department of Medicine, Uddevalla Hospital, Uddevalla, Sweden; 2 Department of Mathematical Sciences, Chalmers University of Technology, Göteborg, Sweden; 3 Department of Medicine, Kungälv Hospital, Kungälv, Sweden; 4 Department of Internal Medicine, Sahlgrenska University Hospital, Gothenburg, Sweden; National Institute of Child Health and Human Development/National Institutes of Health, United States of America

## Abstract

**Aim:**

The updated mean HbA1c has been used in risk estimates of diabetic complications, but it does not take into account the temporal relationship between HbA1c and diabetic complications. We studied whether the updated mean HbA1c underestimated the risk of diabetic complications.

**Method:**

Continuous HbA1c curves for 10,000 hypothetical diabetes patients were simulated over an average of 7 years. Simulations were based on HbA1c values encountered in clinical practice. We assumed that each short time interval of the continuous HbA1c curves had a long-lasting effect on diabetic complications, as evidenced by earlier studies. We tested several different HbA1c variables including various profiles, e.g. different duration, of such a long-lasting effect. The predictive power of these variables was compared with that of the updated mean HbA1c.

**Results:**

The predictive power of the constructed HbA1c variables differed considerably compared to that of the updated mean HbA1c. The risk increase per standard deviation could be almost 100% higher for a constructed predictor than the updated mean HbA1c.

**Conclusions:**

The importance of good glycemic control in preventing diabetic complications could have been underestimated in earlier hallmark studies by not taking the time-dependent effect of HbA1c into account.

## Introduction

Good glycemic control is essential in preventing diabetic complications [1], [2]. The level of glycosylated hemoglobin (HbA1c) provides a measure of the glycemic control of diabetes patients during the previous 2–3 months [3]. Besides the average level of HbA1c, certain changes in HbA1c levels and HbA1c at different points in time can possibly have different implications for the clinician and in studies of the relation between HbA1c and diabetic complications. The term HbA1c-variable is used to describe how different combinations and weighting of HbA1c-values relate to diabetic complications [4].

The HbA1c variable updated mean HbA1c has been used in several hallmark studies of diabetic complications, as it has a greater predictive power than baseline HbA1c [4]–[6]. Updated mean values are often used in the Cox regression model [7], [8]. In such a model, the same importance is given to all the HbA1c values, regardless of when they were measured. This model is thus not suitable when a value or function is expected to both increase and decrease with time. Recent studies indicate that HbA1c levels have a persistent effect on complications several years after their measurement [9]–[12].

In the present study a model was used to determine whether using the updated mean value of HbA1c could substantially underestimate the risk of diabetic complications. As clinical studies of new HbA1c variables require large numbers of patients and the development of new methods, it is important to ascertain whether there is any advantage in using new variables. This can be achieved by simulation. If simulations show that the predictive difference between the updated mean and another predictive variable of HbA1c is small, it will not be necessary to implement changes in clinical practice.

## Methods

### Ethics Statement

The study was approved by the Ethics Committee of the University of Gothenburg.

### Model of analysis

The model of analysis was based on the fact that each diabetes patient has a continuous HbA1c curve. An infinite set of HbA1c variables can be constructed from a continuous HbA1c curve. We assumed that in this infinite set of HbA1c variables there is one “optimal variable”, which takes into account the way in which different levels of HbA1c at different times influence the risk of developing diabetic complications. Contrary to this, the updated mean HbA1c implies that the HbA1c value has the same importance at all points in time. Thus, we constructed HbA1c variables that we believed could be realistic candidates for the optimal HbA1c variable. These variables included scenarios that we believed realistic of how HbA1c during a short time interval affects diabetic complications now and in the future. Hence, no real diabetic complications were used in the model. A mathematical relationship between the predictive power of two variables and the correlation coefficient between them was the basic tool for the comparisons.

The study consisted of three main parts:

1. Simulation of HbA1c values that can be linked together to form a continuous HbA1c curve.
2. Construction of candidates for the optimal HbA1c variable by describing how each short time interval of the continuous HbA1c curves affects diabetic complications now and in the future.
3. Comparison of the predictive power of the HbA1c variables constructed and the updated mean HbA1c.

### Simulation of continuous HbA1c curves

**Figure 1:**
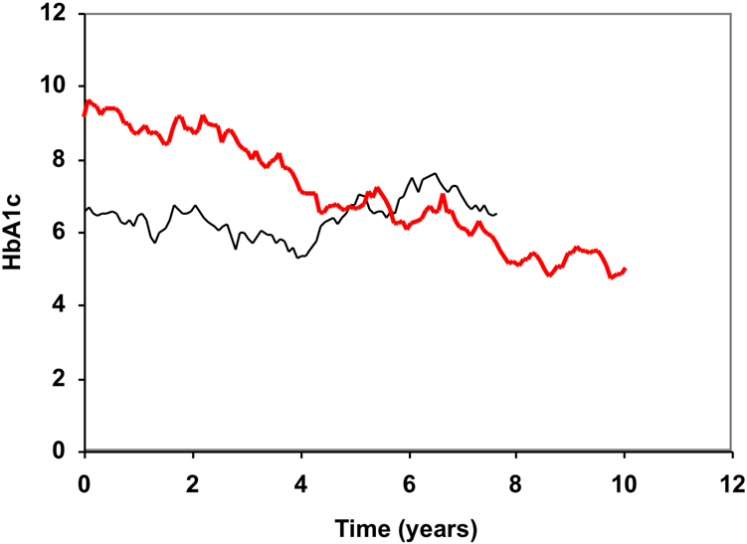
Simulated HbA1c curves. Two examples of simulated HbA1c curves. One of the patients was followed for 7.6 years (black curve) and the other patient for 10.0 years (red curve).

**Figure 2:**
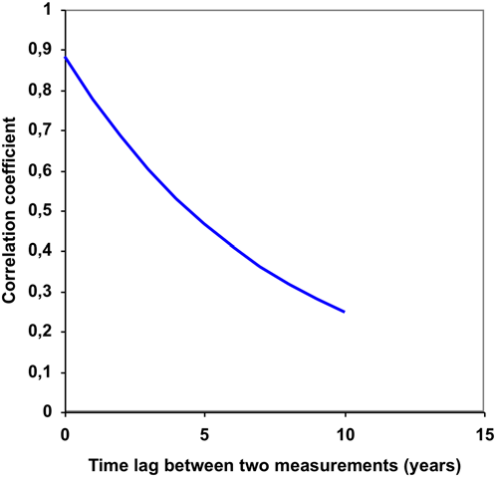
Correlation coefficient between HbA1c values at different points of time. The estimated correlation coefficient between two HbA1c measurements from the same patient, as a function of the time between the measurements.

Continuous HbA1c curves were simulated for 10,000 hypothetical diabetes patients. Monthly HbA1c values were simulated on the basis of HbA1c values from clinical practice and connected by lines to form the continuous curves (Figure 1). The HbA1c measurements from clinical practice were collected from a patient record system called Diab-Base [13]–[15] and were used to determine the correlation coefficient for two values from the same individual as a function of the time interval between them (Figure 2). The coefficient did not differ for type 1 or type 2 diabetes or duration of diabetes. Using this function made the simulations more realistic. The period of the simulated continuous HbA1c curves was varied randomly, and uniformly distributed over the interval 0–14 years, and the average period was 7 years.

We used 65,534 HbA1c values from 12,980 type 1 and type 2 diabetic patients; the mean value of HbA1c was 8.1 (SD 1.3). The values had been obtained by laboratory analysis of HbA1c at local laboratories, with nationwide quality assurance through regular calibration with the high-performance liquid chromatography Mono-S method. In this study, all HbA1c values were converted to the Diabetes Control and Complication Trial (DCCT) standard values using the following formula: A1C (DCCT) = 0.923×A1C (MonoS)+1.345; r^2^ = 0.998 [16].

### Construction of HbA1c variables

We assumed that the maximum harmful effect of HbA1c on diabetic complications is not necessarily manifested at the same time as the current value of HbA1c. We constructed HbA1c variables consisting of an integral of the product of two functions g and f depending on the continuous HbA1c curves (see Supplement S1). The function g reflected how the effect of HbA1c persisted by time and f the relation between the level of HbA1c and diabetic complications.

**Figure 3:**
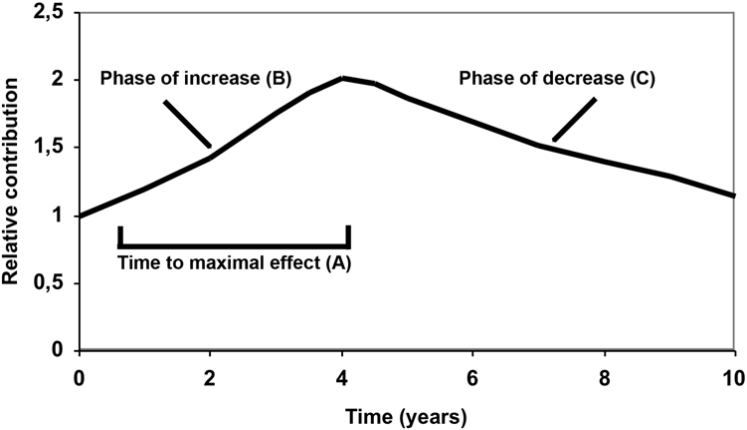
Study model of the temporal relationship between HbA1c and diabetes complications. Relative contribution to the constructed variables at different periods after an HbA1c value was present. The time to maximal effect was A which was reached after a period of increase B and followed by a period of decrease C.

The function g comprised three parameters: 1) time to maximum effect on the development of diabetic complications, 2) the rate of increase in the effect until the maximum is reached, and 3) the rate of decrease in the effect after the maximum (Figure 3).

The persistent effect profiles applied to the simulated continuous HbA1c curves are given in Table 1. Completely flat effect profiles were investigated, i.e. a short interval of HbA1c has a consistent effect on diabetic complications. Profiles with a maximum effect on diabetic complications after 0.5, 2 and 4 years were also tested. The increase in the effect to these maxima was widely varied as was the following decreasing phase.

**Table 1:** The correlation coefficients between the constructed HbA1c variables and the updated mean HbA1c.

Increasing phase (B)	Decreasing phase (C)	Parameter in function f	Correlation coefficients		
Time to doubling (years)	Half life time (years)	Risk increase at higher versus lower HbA1c	Time to maximum = 0.5 years (A)	Time to maximum = 2 years (A)	Time to maximum = 4 years (A)
2	4	20	0.70	0.67	0.62
2	4	1	0.69	0.65	0.56
2	8	1	0.64	0.60	0.53
2	2	1	0.74	0.69	0.61
2	2	8	0.74	0.72	0.66
∞	2	8	0.74	0.73	0.72
1	2	8	0.74	0.71	0.63
2	4	4	0.73	0.70	0.63
4	4	4	0.73	0.71	0.66
4	4	8	0.73	0.71	0.67
1	1	8	0.73	0.71	0.64
2	1	1	0.78	0.73	0.63
∞	1	8	0.73	0.73	0.72
2	4	2	0.71	0.67	0.59
∞	∞	1	0.57	0.57	0.57
∞	∞	8	0.65	0.65	0.65

Columns 1 and 2, together with the headings of columns 4, 5 and 6 characterize the functions g in the constructed variables. The values in the columns 4–6 are the correlation coefficients between the constructed variables and the updated mean HbA1c.

The function f comprised a fourth parameter, which reflected that the risk increase could be different in the interval above 8.7% and 6.0–8.7%. The value 1 of this parameter corresponds to equal risk increase in these two intervals. The function f is defined and illustrated in the Supplement S1.

### Comparison of the predictive power of the updated mean HbA1c value and the HbA1c variables constructed

As a measure of the goodness of the predictors we used the gradient of risk per 1 standard deviation. Due to the existence of a mathematical relationship, which we have derived, between the gradients of two variables and the correlation coefficient between them, we could perform the comparison between the gradients by studying the correlation coefficients between them, see below.

### Statistics

A general measure of the goodness of a predictor is the *gradient of risk per standard deviation*, which is the relative increase in the hazard function when the value of the variable is changed by 1 standard deviation in the direction of risk. This allows comparisons between the goodness of different predictors. The best predictor of the risk of developing a complication based on the complete HbA1c curve during the follow-up period is assumed to be a variable calculated by superimposing an infinite set of curves. Considering one such curve, the corresponding function is assumed to be the product of a function *f* of a single value of HbA1c at time *t* and a function *g* of the time since *t*.

We assume that *f* is continuous everywhere and piece-wise linear. For HbA1c values below 6.0 the function *f* is assumed to be 0, and between 6.0 and 8.7 to increase at a rate *b*. Above 8.7, the rate of increase is assumed to be *b* multiplied by a factor *c*. In the tables below, the factor *c* is referred to as “Parameter in function f”.

The correlation coefficient between a certain HbA1c value and later values was calculated using linear regression. Wiener processes, which have Markovian properties, were used to simulate HbA1c values at monthly intervals [17]. It was assumed that HbA1c values without measurement errors could be well approximated by a Wiener process. For each hypothetical patient we calculated the value of the HbA1c variable as an integral comprising the functions *g* and *f* (see Supplement S1) at the end of the follow-up period; the updated mean was also calculated. The correlation coefficient between the two variables was then calculated. Finally, we applied the relationship given below to compare the gradients of risk.

If a predictor **A** comprises all predictive information that another predictor **B** comprises, then the following relationship between their gradients is true, provided that **A** and **B** have normal distributions:
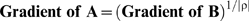
where ρ is the correlation coefficient between A and B.

## Results

**Figure 4:**
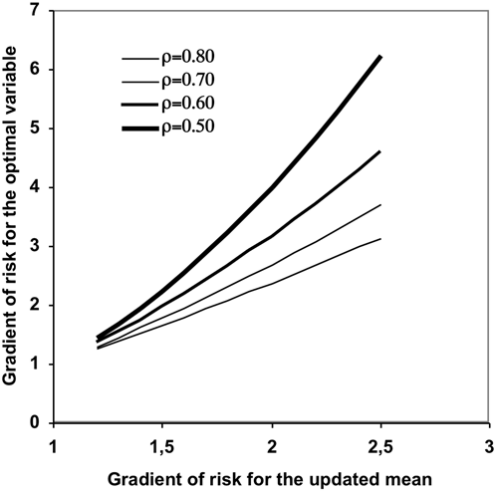
Gradients of risk. Gradient of risk for the updated mean HbA1c and the corresponding estimated gradient for an assumed optimal HbA1c variable. The correlation coefficient between the constructed variables versus the updated mean ranged from 0.53–0.78 and the figure illustrates the cases 0.5, 0.6, 0.7 and 0.8.

The correlation coefficient between the predictive power of the updated mean HbA1c and the constructed variables, which take the time-dependent effect of HbA1c into account, ranged from 0.53 to 0.78 (Table 1). Figure 4 shows the corresponding gradient of risk per SD increase in the constructed HbA1c variables for the correlation coefficients 0.5, 0.6, 0.7 and 0.8, in relation to the gradient of risk per SD for the updated mean HbA1c.

For a certain diabetic complication with a gradient of risk per 1 SD higher updated mean HbA1c of 1.3, a correlation coefficient of 0.5 means that an optimal variable would instead have the gradient 1.69. For another complication, when the gradient is, e.g., 2 per SD higher updated mean HbA1c, a correlation coefficient of 0.5 instead means that an optimal variable would have the gradient 4. A gradient of risk per 1 SD higher of an HbA1c variable of e.g. 1.3 for a certain diabetic complication means that the risk increases by 30% when the HbA1c variable increases 1 SD and a gradient of 4 that the risk increases by 300%.

## Discussion

The practice of using baseline HbA1c in studies on diabetes complications can lead to underestimation of the importance of HbA1c as a risk factor, as only one value is used [4]. Calculation of the updated mean of HbA1c using several values has been found to be better and is widely used [4]–[6]. However, this variable may also result in substantial underestimation of the importance of glycemic control, as the updated mean value of HbA1c gives equal weight to all historical HbA1c measurements. In several of our simulations we found that the increase in risk per standard deviation of an optimal HbA1c variable could be up to twice as high as that predicted by the updated mean HbA1c.

The more complex HbA1c variables investigated here, considering the time-dependent effect of HbA1c, have not been used previously [4]. Although the results of this study are from simulated data, they provide evidence that the updated mean HbA1c is not an optimal variable. The updated mean HbA1c is just one value in an infinite series of possible HbA1c variables, and it has not been constructed empirically [4]. Furthermore, the assumptions in our model of a persistent effect of HbA1c on diabetic complications are strong, whereas there is no evidence that HbA1c values at different points in time will be of the same importance for the development of diabetic complications, which is the case when the updated mean HbA1c is used [4].

The simulated HbA1c curves were based on a result derived from a large number of patients with quality-controlled measurements of HbA1c. The stotchastic procedure used with Wiener processes to simulate the curves has been widely used for randomization procedures [17]. Hence we believe the curves are a good presentation of real diabetic patients in the general population. The time period for the simulated curves was on average 7 years. This could be compared with the Diabetes Control and Complications Trial (DCCT) of 6.5 years, the United Kingdom Prospective Diabetes Study (UKPDS) of 10 years, average duration of type 2 diabetes of roughly 10 years and of type 1 of 25 years [1], [2], [18], [19]. With longer periods of the simulated curves the correlation coefficients would have been even lower and hence the difference of the constructed variables and updated mean HbA1c even greater with respect to predictive power.

The function g in the constructed HbA1c variables reflected how an HbA1c value relates to the risk of developing diabetic complications at the moment and in the future. It can probably not exactly mimic the true relation between HbA1c and a certain diabetic complication. It is only composed of three parameters, but these make it possible to vary how fast the effect increases and decreases, when the maximum is reached, and can in a rough way mimic all possible and likely scenarios. The real curve might however be more smooth e.g. at its peak and shapes of the slopes. The function was widely varied so that the effect had peak at different points of time and the time until the effect became less than the initial effect varied from 0.75 to 20 years.

Evidence of a persistent effect of HbA1c such as those used in the constructed variables has been presented in the Epidemiology of Diabetes Interventions and Complications (EDIC) studies [9]–[11], as well as in the recently presented follow-up of the UKPDS [12]. The EDIC study shows that the HbA1c level over a period of 6.5 years is of the same importance during the next 4 years regarding the development of retinopathy, and for the next 8 years concerning micro- and macroalbuminuria [9]–[10]. Concerning myocardial infarction and stroke, it was shown that the values had a substantial average influence during the next 11-year period, although it was not shown how the effect varied during this period [11]. In the post-UKPDS study, the effect of intensive treatment did not become evident until the 10-year follow-up after the end of randomization [12]. Other studies also support the persistent effect of HbA1c, showing that the maximum effect on complications is probably not exhibited when a specific HbA1c value is measured, but rather some years later [2], [4], [9], [10], [20].

The function f, included in the constructed HbA1c variables, was introduced to allow different risk increase per unit of HbA1c in different intervals. The updated mean HbA1c does not allow different increase of the risk per unit HbA1c. For several examples of constructed variables we let this relation be similar as for the updated mean HbA1c. Then mainly the persistent effect of HbA1c reflected by the function g in the constructed variables differed from the updated mean HbA1c. For other constructed variables the relative risk increase for one unit higher HbA1c was greater above HbA1c 8.7%. The predictive power differed substantially, as reflected by the correlation coefficient, for all constructed variables compared to up-dated mean HbA1c. Hence the persistent effect of HbA1c had a strong influence on the difference in predictive power.

Knowledge about the relationship between HbA1c and the risk of diabetic complications is important when evaluating expensive forms of treatment [21]. Small effects of treatment on HbA1c can easily be overlooked. The cost of treating diabetes patients constitutes a large part of the total health care budget, and it is thus important from the economic perspective to have a sound knowledge of the effects of glycemic control on complications [22]–[24]. The risk engines in use today are based on a mean value of two measurements, and the risk of a particular complication will thus probably be much lower than that given by an optimal variable [25]. This could lead to inappropriate decisions by both the clinician and the patient.

Underestimating the role of a risk factor may also lead to incorrect conclusions regarding etiology. Since the updated mean is employed in the widely used Cox regression model, the underestimation of risk factors may be common in other medical fields. Using an optimal predictor would, for example, be of importance when correlating blood lipids and blood pressure to stroke and myocardial infarction [26]–[27].

The role of different risk factors in medicine in general could probably be better assessed by studying the correlation between measurements of the risk factor at different points in time and the outcome. The model of the constructed variables presented here could be used by an optimization procedure determining the functions g and f for an optimally predictive variable. Patient materials with frequent measurements of HbA1c and evaluations of diabetic complications would be preferable to use. The function g will reflect how long time it takes until an improvement in glycemic control becomes salatory in preventing diabetic complications. Hence, besides a more accurate estimation of the risk gradient between HbA1c and diabetic complications the presented method can be of importance in the clinic for prognosis and pathogenesis understanding which HbA1c values in time relate to any developed diabetic complications. In the design of clinical trials knowledge of the temporal relationship between HbA1c and diabetic complications is essential so that an appropriate study length is chosen and an improvement in glycemic control can lead to beneficial effects on diabetic complications.

## Supporting Information

Supplement S1 Supplementary Material
(0.03 MB DOC)
