# Supplementary material for: The True Value of HbA1c as a Predictor of Diabetic Complications: Simulations of HbA1c Variables
Source: PLoS One. 2009 Feb 11;4(2):e4412. doi: 10.1371/journal.pone.0004412 (PMC2636883; doi:10.1371/journal.pone.0004412)
Supplement: Supplement S1 — Supplementary Material (0.03 MB DOC) [file pone.0004412.s001.doc]

2006-08-21

**Supplement 1**

**The functions f and g**

A general measure of the goodness of a predictor is *gradient of risk per 1 standard deviation*,

which is the relative increase of the hazard function when the value of the variable is changed

1 standard deviation in the direction of risk. The measure allows us to compare the goodness

of different predictors. The best predictor of the risk for complication based on the complete

curve of HbA1c from diagnosis and onwards is assumed to be a variable calculated by

considering superimpositions of an infinite set of curves. Let us consider one such curve. The

corresponding function is assumed to be a product between a function f of a single value of

HbA1c at time t and a function g of time since t.

We assume that f is continuous everywhere and piece wise linear. For HbA1c values below 5

the function f is assumed to be 0 and between 5 and 8 increasing with a slope b and above 8

with a slope b times a factor c. The factor c is in table 1 referred to as “Parameter in function

f”.

**The function f** We assume that the function g is everywhere continuous and exponentially increasing since

the time t until a certain time period, which in our different simulations are put to 0.5, 2 and 4

years, respectively. After that time period g is assumed to be exponentially decreasing. We

have chosen some different rates of increase corresponding to doubling in 1, 2, 4 and ∞

years. Similarly for decrease we have chosen the rates for reaching half of a previous value to

1, 2, 4, 8 and ∞ years.

**The function g** If z denotes the time since t then the product between g and f can be written as

exp((log(2)/2)·min(z, 2)+(log(.5)/4)·max(z-2, 0)) · (b·max(min(HbA1c-5, 8-5), 0)+b·

c·max(HbA1c-8, 0))

where the rate of increase corresponds to time to doubling of 2 years, the rate of increase

corresponds to a half life time of 4 and the time to reaching the maximum is 2 years. The total

contribution to the risk at time t is given by:

*f v g t v dv*

*t*∫

⋅ −

0

( ) ( ) , which constitutes the constructed variables.
